# Keypoints-Based Multi-Cue Feature Fusion Network (MF-Net) for Action Recognition of ADHD Children in TOVA Assessment

**DOI:** 10.3390/bioengineering11121210

**Published:** 2024-11-29

**Authors:** Wanyu Tang, Chao Shi, Yuanyuan Li, Zhonglan Tang, Gang Yang, Jing Zhang, Ling He

**Affiliations:** 1 College of Biomedical Engineering, Sichuan University, Chengdu 610065, China; 2022223100045@stu.scu.edu.cn (W.T.); cs9387476@gmail.com (C.S.); tang.zhonglan@scu.edu.cn (Z.T.); yang_gang@scu.edu.cn (G.Y.); jing_zhang@scu.edu.cn (J.Z.); 2 Mental Health Center, West China School of Medicine, Sichuan University, Chengdu 610041, China; guojipangxie@126.com

**Keywords:** attention deficit hyperactivity disorder, keypoints-based action recognition, graph neural network, multi-cue feature fusion

## Abstract

Attention deficit hyperactivity disorder (ADHD) is a prevalent neurodevelopmental disorder among children and adolescents. Behavioral detection and analysis play a crucial role in ADHD diagnosis and assessment by objectively quantifying hyperactivity and impulsivity symptoms. Existing video-based action recognition algorithms focus on object or interpersonal interactions, they may overlook ADHD-specific behaviors. Current keypoints-based algorithms, although effective in attenuating environmental interference, struggle to accurately model the sudden and irregular movements characteristic of ADHD children. This work proposes a novel keypoints-based system, the Multi-cue Feature Fusion Network (MF-Net), for recognizing actions and behaviors of children with ADHD during the Test of Variables of Attention (TOVA). The system aims to assess ADHD symptoms as described in the DSM-V by extracting features from human body and facial keypoints. For human body keypoints, we introduce the Multi-scale Features and Frame-Attention Adaptive Graph Convolutional Network (MSF-AGCN) to extract irregular and impulsive motion features. For facial keypoints, we transform data into images and employ MobileVitv2 for transfer learning to capture facial and head movement features. Ultimately, a feature fusion module is designed to fuse the features from both branches, yielding the final action category prediction. The system, evaluated on 3801 video samples of ADHD children, achieves 90.6% top-1 accuracy and 97.6% top-2 accuracy across six action categories. Additional validation experiments on public datasets NW-UCLA, NTU-2D, and AFEW-VA verify the network’s performance.

## 1. Introduction

**Figure 1:**
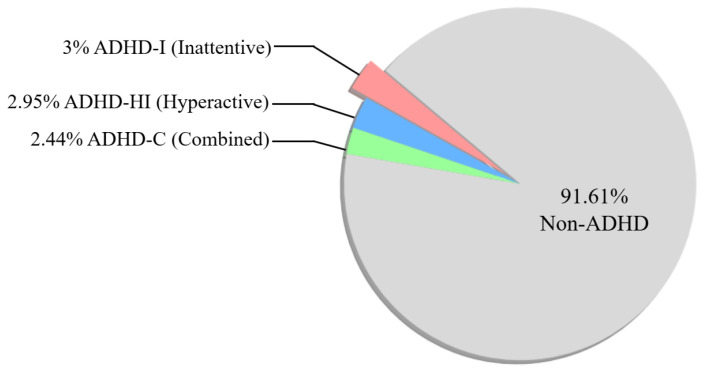
The pie chart of ADHD prevalence statistics. The total sample size is 3,277,590, which includes data from various studies conducted between 2007 and 2022. The overall prevalence rate is 8%. The prevalence rates for each subtype are as follows: ADHD-I (3%), ADHD-HI (2.95%), and ADHD-C (2.44%).

Attention Deficit Hyperactivity Disorder (ADHD) is one of the prevalent neurodevelopmental disorders in childhood [1,2]. It is characterized by a range of symptoms, including inattention, hyperactivity, and impulsivity [1]. According to a survey [3] compiling data from various studies, involving a total sample of 3277590 participants, ADHD in children and adolescents was 8.0% (all figures represent statistically verified results). The inattentive type of ADHD was the commonest type of ADHD (ADHD-I) 3% followed by hyperactive type (ADHD-HI) 2.95% and the combined type (ADHD-C) 2.44%, as shown in Figure 1. These symptoms may place significant stress on the learning and interpersonal interactions of children with ADHD, while also adversely affecting their mental health and increasing the risk of developing depression and anxiety disorders. Furthermore, children with ADHD may encounter challenges in employment as they transition into adulthood. These symptoms can adversely affect their learning and daily life, and may persist into adulthood [4,5,6,7,8,9]. This underscores the necessity of early detection and treatment for children with ADHD. Clinically, the diagnosis of ADHD is often based on subjective evaluations by healthcare professionals. This typically involves using standardized criteria such as the DSM-V or interviewing about the child’s daily behavior [10].

The inherent variability in human observation and interpretation presents considerable challenges in achieving consistent diagnoses across different environments and evaluators. Objective measurements can reveal subtle behavioral patterns or symptom manifestations that may not be easily observed through traditional assessment methods, thereby enriching our understanding of ADHD. In addition to diagnosis through the DSM-5, several studies have explored the causes and symptoms of ADHD, proposing objective indicators and methods to identify the disorder. These methods include: (1) Brain activity and physiological signal detection, such as functional magnetic resonance imaging (fMRI) [11,12,13], electroencephalography (EEG) [14]. While these techniques provide valuable insights into brain function, they are often expensive, time-consuming, and require specialized equipment and expertise, limiting their widespread application in clinical settings. (2) Behavioral detection and analysis, such as handwriting analysis [15], body movement analysis during sleep [16,17] and wearable device detection system [18,19,20,21,22]. Although these methods offer less invasive alternatives, they may not reliably capture the prominent symptoms needed for accurate diagnosis. Furthermore, wearable devices can potentially inconvenience children with ADHD, inadvertently restricting their natural activities and potentially skewing results.

In addition to the aforementioned methods, Test of Variables of Attention (TOVA) is another widely employed clinical tool for the diagnosis of ADHD in children. It is specifically designed to assess children’s attention and impulsivity levels [23]. Its measurement approach focuses on assessing individual attention deficits during task performance, including sustained and selective attention, aiding clinicians in accurately evaluating patients’ attention levels. It has become commonly used to diagnose the severity of ADHD in children [24,25,26,27,28]. TOVA has been validated as a reliable method in many studies [29,30,31,32,33,34]. It demonstrates objectivity and is less influenced by confounding variables. Compared to the use of expensive and complex equipment, TOVA provides objective results concerning ADHD attention levels with a straightforward test. During the test, children often exhibit restless behaviors due to attention deficits or hyperactivity and impulsivity. Analyzing the behavioral performance during the TOVA can serve as an assessment of ADHD symptoms as described in the DSM-V. This can provide additional data points to support clinical judgment, potentially enhancing the accuracy and consistency of diagnoses. Therefore, we propose a novel and reliable paradigm for analyzing the behavior of children with ADHD to aid in the analysis of TOVA results, which utilizes cameras to record and recognize children’s actions during the TOVA.

Action recognition in computer vision has traditionally focused on a wide range of tasks involving complex interactions with objects or other individuals, such as drinking water or shaking hands. Established action recognition networks, including Two-stream CNN [35], 3D-ResNet [36], and various multimodal networks [37,38], have been designed to process not only human movements but also the contextual information provided by objects and environments. These approaches have demonstrated considerable success in general action recognition scenarios where the interplay between humans and their surroundings is crucial for accurate classification.

However, the specific requirements of our research, centered on the Test of Variables of Attention (TOVA) for ADHD diagnosis, present a unique set of challenges that diverge from conventional action recognition tasks. The behaviors exhibited during TOVA primarily consist of intricate combinations of movements from various body parts, with minimal relevance of background elements. This distinctive characteristic of our task necessitates a shift in focus towards the individual undergoing assessment, emphasizing the importance of precise body part localization and sophisticated motion modeling. In light of these requirements, our approach aligns more closely with methods that prioritize human-centric data. Graph Convolutional Neural Networks (GCNs) based on skeleton data, such as ST-GCN [39] and 2s-AGCN [40], have emerged as prominent solutions in action recognition tasks that benefit from isolating human movement from environmental factors. These methods leverage extracted human body keypoint information to construct graph-based representations of human poses and movements over time, effectively eliminating external environmental interference, offering a promising foundation for our ADHD-focused action recognition task.

Nevertheless, the application of existing GCN-based methods to our specific context reveals certain limitations. Children with ADHD undergoing TOVA often exhibit sudden, unpredictable movements, such as abruptly shifting their body position while resting on a table. These sporadic behaviors pose certain challenges to traditional GCN approaches, which typically model movements over entire time sequences. Such methods may fail to capture the significance of critical frames or adequately represent local temporal movements that are crucial for identifying ADHD-related behaviors. Additionally, while previous GCN-based networks have not extensively utilized facial keypoints due to their limited contribution to action recognition in other tasks, our research recognizes the unique importance of facial and head movements in ADHD assessment. Children with ADHD often display distinctive facial expressions and head movements during TOVA, making the integration of facial keypoints crucial for comprehensive motion modeling in our context.

To address these ADHD-specific challenges, we propose a Multi-cue Feature Fusion Network (MF-Net). While our framework builds upon established foundations in action recognition, it introduces several key innovations specifically designed for ADHD behavior analysis: For human keypoints data: (1) First, we develop a novel Multi-scale Spatial-Temporal Information Extraction Module integrated with AGCN. Unlike existing multi-scale approaches that focus on general action recognition, our module specifically targets the temporal irregularity of ADHD movements through an adaptive feature extraction mechanism. This innovation enables the simultaneous capture of both sustained attention patterns and sudden impulsive movements - a capability crucial for ADHD assessment but not addressed by current methods. (2) Second, we design an innovative Motion Attribute Encoder Module that substantially advances CBAM’s temporal attention mechanism by introducing a new temporal channel encoding mechanism to identify and emphasize frames containing characteristic ADHD behaviors, enabling more precise capture of patients’ irregular movement patterns. This module innovatively identifies and emphasizes frames containing diagnostically relevant movements, specifically addressing the challenge of detecting ADHD-related behavioral patterns. The integration with facial keypoint analysis through our adapted MobileViTv2 network further enhances our system’s ability to capture comprehensive behavioral characteristics.

Our proposed Multi-cue Feature Fusion Network (MF-Net) offers a novel, objective approach to quantifying hyperactivity and impulsivity symptoms during the Test of Variables of Attention (TOVA), potentially enhancing the accuracy and efficiency of ADHD diagnosis. This multifaceted approach not only advances the technical aspects of action recognition but also demonstrates the potential for AI-assisted clinical tools in neurodevelopmental disorder assessment.

In summary, our main contributions are as follows:

- We present a novel multi-cue framework that effectively integrates both global body movements and local facial-head movements through a novel fusion strategy, achieving superior performance in ADHD behavior recognition during TOVA assessment.
- We propose two innovative modules for ADHD-specific feature extraction: a Multi-scale Spatial-Temporal Information Extraction Module that captures movement patterns at different temporal scales, and a Motion Attribute Encoder that identifies critical behavioral frames. These modules work synergistically to model both sustained attention and sudden impulsive movements characteristic of ADHD.
- We present a MobileViTv2 classification network that constructs images based on facial keypoints.

## 2. Methods

### 2.1. Overview of MF-Net

**Figure 2:**
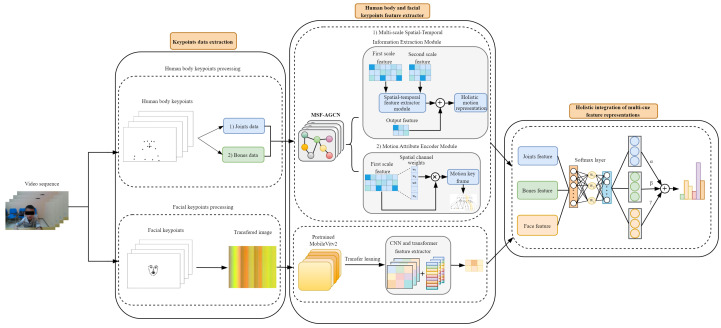
The overall framework proposed in this paper. The system encompasses three components: Keypoints data extraction, Human body and facial keypoints feature extractor, and Holistic integration of multi-cue feature representations. The feature extraction process of MF-Net comprises three branches: MSF-AGCN (joints) for modeling joint movements, MSF-AGCN (bones) for capturing skeletal deformations, and Mobilevitv2 (face) dedicated to extracting facial keypoints features.

In order to recognize the erratic, brief, and rapid actions displayed by children with ADHD during the TOVA, we propose a Multi-cue Feature Fusion Network (MF-Net) by extracting motion features from both body keypoints and facial keypoints. For the global body keypoints feature, a Multi-scale Features and Frame-Attention Adaptive Graph Convolutional Network (MSF-AGCN) is constructed for extracting features from both joints and bones data. The network adopts AGCN as the backbone network. In this network, two innovative modules are proposed: Multi-scale Spatial-Temporal Information Extraction Module and Motion Attribute Encoder Module. The Multi-scale Spatial-Temporal Information Extraction Module (MSST ) performs attention-based feature extraction at different dimensions of the backbone network and is fused with the final output of the backbone network. The Motion Attribute Encoder Module (MAE) is utilized for temporal-dimension attention extraction in the early stage of the backbone network. For the local facial keypoints feature, facial keypoints data from each sample are converted into pseudo-color images and processed using MobileVitv2 in transfer learning to capture fine-grained facial and head movements. Finally, the extracted features from body keypoints and facial keypoints are fused to obtain action classification results. An overview of the complete method is illustrated in the Figure 2.

### 2.2. Human Body Keypoints Feature Extractor

#### 2.2.1. Adaptive Graph Convolutional Network

The actions defined involve the motion states of various body parts exhibited by children with ADHD during the TOVA. The Graph Convolutional Network (GCN) constructed from body keypoints not only localizes individual parts but also encodes continuous motion information. Spatial-Temporal Graph Convolutional Network (ST-GCN) introduces the application of GCN to skeleton-based action recognition. This network simply defines the graph in graph convolution and is inadequate for complex motion modeling. Adaptive Graph Convolutional Network (AGCN) adopted in this feature extractor is an improved graph convolutional network for holistic action modeling based on skeleton data.

**Figure 3:**
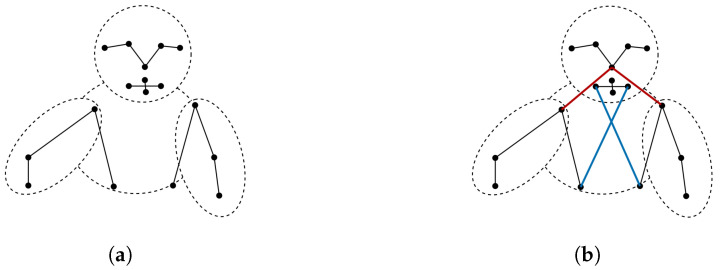
Two different graph connections. (**a**) New Graph. This excludes the connections of the nose, mouth, and body. (**b**) Our Graph. This includes additional connections, where the red lines represent the connections between the nose and shoulders, and the blue lines represent the connections between the mouth and body.

The core component of AGCN is the G-TCN module. It consists of graph convolution operations and temporal convolution operations. Graph convolution operations consist of two main parts: the construction of the graph and the method of performing graph convolutions. For the first part, graph convolutions extract features from the skeleton graph G=(V,E), where $V=\left\{v_{i,t},i=1,2\ldots N,t=1,2\ldots T\right\}$ represents the set of keypoints, and $E=(E_{frame\_within},E_{frame\_inter})$ represents the set of edges connecting the keypoints. The edge set includes $E_{frame\_within}$, connections between different keypoints within the same frame, as well as $E_{frame\_inter}$, connections between the same keypoints across different frames. The edges capture temporal evolution, connecting keypoints within frames and across frames. In the $E_{frame\_within}$, unlike connections limited to adjacent joints, we add two different types of connections as shown in Figure 3b: (1) connections between the nose and shoulders to enhance detection of head movements relative to the body, aiding in recognizing behaviors such as sudden head turns; (2) connections between the left and right hip keypoints and the mouth keypoints, shortening the topological distance between the face and body. This facilitates the capture of body swaying motion features.

**Figure 4:**
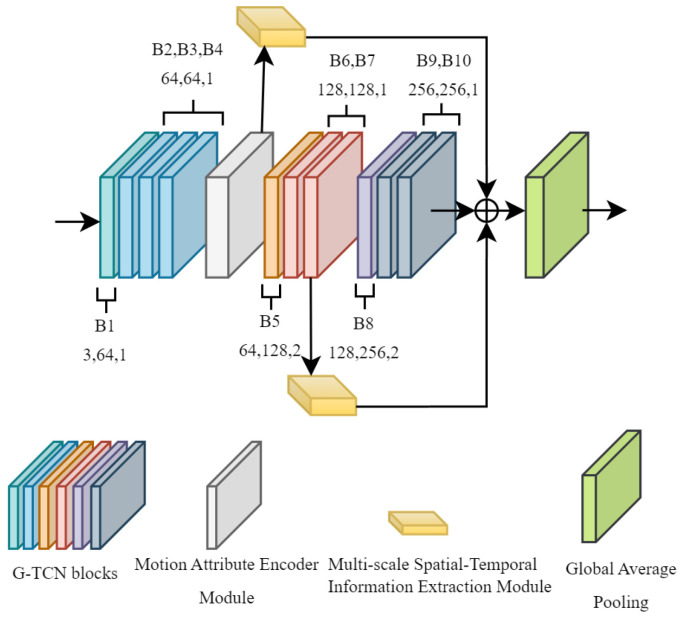
The structure of MSF-AGCN. The network architecture is built upon the Adaptive Graph Convolutional Network (AGCN) as its baseline, incorporating two novel components: the Multi-scale Spatial-Temporal Information Extraction Module and the Motion Attribute Encoder Module.

For the second part, AGCN introduces an attention mechanism when computing the graph convolution. The output of the graph convolution can be represented as:

(1) $$f_{out}=\sum_{k}^{K_{v}}W_{k}f_{in}\left(A_{k}+B_{k}+C_{k}\right)$$

where $f_{in}$ represents a spatial-temporal graph constructed from input keypoints data, $W_{k}$ is the weight vector obtained through 1×1 convolution, and $A_{k}$ is adjacency matrix defined between keypoints in the first part, $B_{k}$ is adjacency matrix with learnable parameters that are initialized, and $C_{k}$ is adjacency matrix learned through the attention mechanism. However, its reliance on global temporal modeling often fails to account for the importance of critical frames and localized temporal features. This structural limitation hinders the network’s ability to effectively capture the nuanced and abrupt motion patterns that are characteristic of ADHD children’s behaviors. To this end, we construct Multi-scale Features and Frame-Attention Adaptive Graph Convolutional Network (MSF-AGCN), as illustrated in Figure 4. The following two modules are proposed for enhancements.

#### 2.2.2. Multi-Scale Spatial-Temporal Information Extraction Module

The module aims to model the brief and rapid actions (e.g., sudden turning, lying down) as well as actions with varying amplitudes (e.g., slight head turning, vigorous body swaying) exhibited by children with ADHD during the TOVA at different scales. It takes into account the influence of local motion information on the overall motion state.

**Figure 5:**
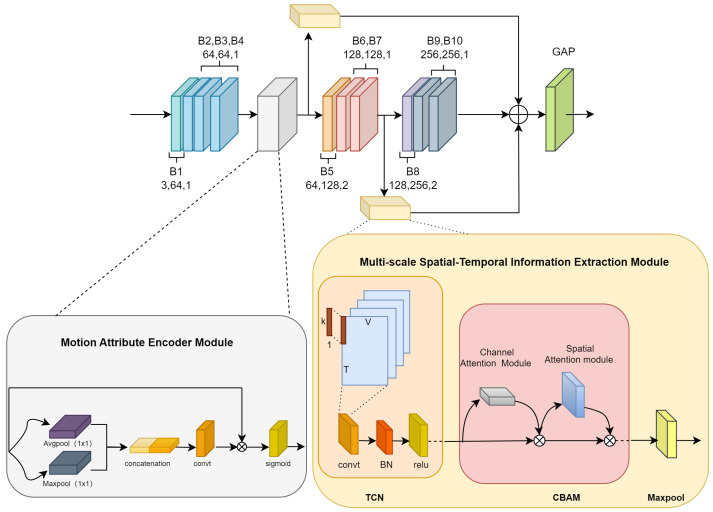
The architectures of the Multi-scale Spatial-Temporal Information Extraction Module and the Motion Attribute Encoder Module proposed in the MSF-AGCN.

As shown in Figure 5, this module consists of three parts: Temporal Convolutional Network (TCN) operation, Convolutional Block Attention Module (CBAM) [41] and MaxPool layer.

(1) TCN operation, which is used to learn the motion patterns and dynamic information of the keypoints data over time from the low-level features of the input. It first employs a temporal convolution with a kernel size of 9×1 along the temporal dimension. The temporal convolution operation changes the number of channels of the output features, thereby extracting more advanced and abstract feature representations in the temporal dimension. Subsequently, the BatchNormalization layer is applied to normalize the output features of the temporal convolution. Finally, the ReLU activation function is used to achieve non-linear mapping. This operation can be expressed by the following formula:

(2) $$X^{\left(l+1\right)}=ReLU\left(Batch\;Norm\left(conv\left(X^{l},W_{0}\right)\right)\right)$$

where $X^{l}$, $X^{\left(l+1\right)}$ represents the lth, (l+1)th features, respectively. $W_{0}$ represents the convolutional kernel weights with a kernel size of 9×1, conv represents the temporal convolution operation.

(2) Convolutional Block Attention Module (CBAM). This module consists of two sub-modules: the channel attention module and the temporal attention module. Two modules are concatenated and executed sequentially. The channel attention module aims to adaptively adjust the importance weights of different feature channels to highlight the more relevant and discriminative feature channels for the current task. The temporal attention module focuses on the attention allocation at different time steps, enhancing the modeling capability of key time segments. Its operation is shown in the following formula:

(3) $$X^{l+2}=X^{l+1}⊙M_{c}\left(X^{l+1}\right)⊙M_{s}\left(X^{l+1}\right)$$

where $X^{l+2}$ represents the the feature output by the CBAM module. $M_{c}\left(X^{l+1}\right)$ and $M_{s}\left(X^{l+1}\right)$ denote the channel attention module and temporal attention module, respectively. The ⊙ represents element-wise multiplication.

(3) MaxPool layer. This layer is applied to compress the size of the feature maps and capture the most prominent feature information within the local region. The maxpool operation is formulated as:

(4) $${X_{i,j}}^{l+3,k}=\max_{\left(m,n\right)\in R_{i,j}}{X_{m,n}}^{l+2,k}$$

where ${X_{m,n}}^{l+2,k}$, ${X_{i,j}}^{l+3,k}$ are the features before and after maxpool, respectively.

Assuming the input feature size is $X^{l}\in R^{C\times T\times V}$, where *C* denotes channel, *T* denotes frames and *V* denotes joints, after processing by Multi-scale Spatial-Temporal Information Extraction module, the output feature shape becomes $X^{l+3}\in R^{C_{out}\times T_{out}\times V}$, This module is placed after the outputs of the fourth and seventh blocks of the network to extract keypoints features at the first and second stages of the temporal scale, respectively. These features are then fused with the final output features of AGCN, resulting in an enhanced output feature representation. The final output can be represented by the following formula:

(5) $$F={X_{4}}^{l+3}+{X_{7}}^{l+3}+X_{10}$$

where ${X_{4}}^{l+3}$, ${X_{7}}^{l+3}$, $X_{10}$ represent the spatial-temporal features extracted after the 4th block, the spatial-temporal features extracted after the 7th block, and the output features from the 10th block in AGCN, respectively.

#### 2.2.3. Motion Attribute Encoder Module

This module aims to extract temporal attention features to address the challenges of modeling irregular motion patterns. The design of this module modifies certain aspects of the Convolutional Block Attention Module (CBAM) attention mechanism, incorporating attention to extract temporal features along the time dimension. As shown in Figure 5, the dimensions *C* and *T* of the input feature $X^{l}\in R^{C\times T\times V}$ are swapped to $X^{l_{1}}\in R^{T\times C\times V}$ using a transpose operation. Then the adjusted features are subjected to adaptive 1×1 average pooling and max pooling operations on the last two dimensions, respectively. These pooled features are then merged into a fused, dimension-reduced feature $X^{l_{2}}\in R^{T\times 2\times 1}$ via a concatenation operation. This process can be formulated as:

(6) $$X^{l_{2}}=\left[avgpool\left(X^{l_{1}},\left(1,1\right)\right),maxpool\left(X^{l_{1}},\left(1,1\right)\right),axis=1\right]$$

where $avgpool(X^{l_{1}},\left(1,1\right))$, $maxpool(X^{l_{1}},\left(1,1\right))$ denote using 1×1 feature maps for adaptive average pooling and max pooling, with axis=1 indicating the operation is performed along the second dimension. After that, a temporal convolution layer with a kernel size of (9, 1) and a stride of 2 is applied to further extract the attention scores, followed by activation using the sigmoid function. This results in an attention score feature $X^{l_{3}}\in R^{T\times 1\times 1}$. Finally, the attention scores along the *T* dimension are multiplied with the original input feature to obtain the output features. It generates a tensor representation that encodes the temporal attention information of the input feature. The output is formulated as:

(7) $$X^{l_{4}}=X^{l}⊗\sigma (conv\left(X^{l_{2}},W_{1}\right)$$

where σ denotes the sigmoid activation function, while $W_{1}$ denotes the convolutional kernel weights with a kernel size of 9×1.

The Motion Attribute Encoder module is placed after the 4th block of the network to extract temporal attention features at an early stage across all time frames, capturing the motion attributes of the entire sequence.

### 2.3. Facial Keypoints Feature Extractor

#### 2.3.1. Transformation of Facial Keypoints into Images

Facial keypoints can capture head movements and facial expression changes, especially in the movements exhibited by ADHD children during the TOVA. Considering the weaker connectivity between facial keypoints compared to skeleton keypoints, constructing a graph structure is not suitable. Furthermore, the overall facial configuration during motion can reveal subtle movements. Therefore, we transform facial keypoints into images as input to extract features. We extracted 68 facial keypoints for each frame. Each keypoint $P_{i,j}$ for the ith frame and jth keypoint is represented by its coordinates $\left(X_{i,j},Y_{i,j}\right)$ and a confidence score $S_{i,j}$. To prepare the data for visualization, the coordinates and scores are normalized to fit within the RGB color range [0,255]. The coordinates are scaled based on the maximum values observed in the dataset, $X_{max}$ and $Y_{max}$, across all keypoints. The normalized coordinates are computed as follows:

(8) $${X^{\prime }}_{i,j}=\left|\frac{X_{i,j}}{X_{\max}}\right|\times 255,{Y^{\prime }}_{i,j}=\left|\frac{Y_{i,j}}{Y_{\max}}\right|\times 255$$

Similarly, the confidence scores $S_{i,j}$, which inheretly lie between 0 and 1, are directly scaled to the range [0,255]:

(9) $${S^{\prime }}_{i,j}=S_{i,j}\times 255$$

Finally, image *I* of dimensions 68×60 pixels is constructed. The RGB value of each pixel in the image is set based on the normalized values:

(10) $$I_{i,j}=\left({X^{\prime }}_{i,j},{Y^{\prime }}_{i,j},{S^{\prime }}_{i,j}\right)$$

#### 2.3.2. Feature Extraction on Transformed Images

After the above processing, we obtain the new input data $I\in R^{C\times H\times W}$. MobileViTv2 is employed for the extraction of data features. It is a variant of MobileViT, a lightweight model that combines CNN and Transformer to reduce parameter count. MobileViTv2 not only has the ability to capture image information but also possesses strong global sequence modeling capabilities due to the Transformer used, as the input data contains sequential relationships. We retain the main network of MobileViTv2 and modify the output of the final fully connected layer. It can be expressed as:

(11) $$Y=W_{2}\cdot MobileViTv2_{main}\left(I\right)+b$$

where $W_{2}$ represents the weights of the fully connected layer.

### 2.4. Holistic Integration of Multi-Cue Feature Representations

For the task of recognizing behaviors in children with ADHD, motion cues from body joints, body part movements, and facial expressions and head motions serve as important complementary sources of information. Our proposed MF-Net model aims to comprehensively leverage these diverse feature modalities. Specifically, the MSF-AGCN module extracts global motion features $F_{joints}$ and $F_{bones}$ from human body keypoints representing joint and bone movements, respectively. The MobileViTv2 focuses on local facial keypoints to learn facial motion features $F_{face}$. These three feature sources encompass distinct yet complementary types of information. To effectively fuse these multi cue features, we design a feature fusion module that performs weighted fusion of the outputs from the MSF-AGCN and MobileViTv2 branches, generating a holistic feature representation for behavior classification. This module first applies softmax processing to the fully connected layer outputs of the individual branches, yielding probability distribution tensors. Subsequently, it adjusts the weights of these tensors and combines them to obtain the optimal behavior recognition result. This process can be expressed as:

(12) $$F_{1}=W_{3}\cdot \psi \left(F_{joints}\right)+W_{4}\cdot \psi \left(F_{bones}\right)+W_{5}\cdot \psi \left(F_{face}\right)$$

where ψ denotes the softmax function, and $W_{3}$, $W_{4}$, $W_{5}$ denote the feature weights for the joints, bones, and face branches, respectively.

## 3. Experiments

### 3.1. Datasets

SCU-ADHD-Action-Dataset. The TOVA visual test spans a total of 21.6 min. During the test, children sat in front of a computer screen that flashed every two seconds, with a black square appearing either above or below the white area on the screen. When the black square appeared above, the children were required to click the left mouse button. Cameras were placed above the computer screen and under the desk to record the children’s full-body movements from the start of the test. As the lower body movements were not significant, only the data from the top camera was used in this experiment. For each child, 20 min of valid video data were extracted and segmented into individual samples based on the screen flash interval (every two seconds), with each sample containing 60 frames. The size of each frame image is 1280 (width) × 720 (height) pixels. In this work, we collected video data of 7 ADHD children during the TOVA visual test at the Mental Health Center of West China Hospital, Sichuan University. After data screening and segmentation, a total of 3801 video samples were obtained.

By detecting the keypoints of the children in each frame of every video, the keypoints information is obtained, forming a time-series keypoints data sample. To obtain comprehensive keypoint information, we employ AlphaPose [42,43,44], a state-of-the-art tool renowned for its accuracy and versatility in human pose estimation. AlphaPose is particularly suitable for our research due to its ability to extract both body and facial keypoints simultaneously, a feature critical for our holistic approach to ADHD behavior analysis. The keypoint extraction process is applied to each frame of every video in our dataset, resulting in a time-series of keypoints data. This approach allows us to capture the dynamic nature of children’s movements throughout the TOVA, providing a rich dataset for subsequent analysis. The keypoints data extracted by AlphaPose for a single frame is a three-dimensional vector (x,y,s), where *x* and *y* represent the coordinates of the keypoint in the frame, and *s* indicates the confidence score. The keypoints data for each video sample has a shape of (C,T,V), where *C* represents the three dimensions of a single keypoint data, *T* is the number of frames in the video, and *V* is the number of joints. We incorporate 17 key body points, comprising: 9 head keypoints: These include crucial keypoints such as the eyes, ears, and nose, allowing us to track head movements and orientation, which are often indicative of attention shifts and impulsivity; 8 upper body keypoints: Focusing on the shoulders, arms, and upper torso, these points enable us to capture gross motor movements and postural changes frequently observed in children with ADHD. We also extract 68 facial keypoints, which provide granular facial data that is particularly valuable for detecting subtle cues of inattention or facial states that may not be apparent from body movements alone. To ensure the reliability and consistency of our keypoint data, we implement preprocessing steps focused on missing data handling; specifically, we employ interpolation techniques to estimate the positions of occluded or low-confidence keypoints based on surrounding frames, ensuring continuity in our time-series data.

Based on clinical expertise, we define six action state categories that characterize the behavioral manifestations of children with ADHD during the TOVA visual test, as detailed below:

(1) Turning the head and look around. This action manifests when the child is unable to concentrate. Their attention is diverted by the surrounding environment, leading to sudden and unpredictable behavior. It is indicative of symptoms of inattention.
(2) Shaking body. This behavior arises from symptoms of hyperactivity and impulsivity. It leads to an inability to sustain attention on a single task for an extended period. It is manifested as body movements while seated, characterized by irregularity.
(3) Resting head on hand. This action occurs when the child feels fatigued or has difficulty maintaining attention. It is related to symptoms of inattention.
(4) Displaying facial expressions of inattention. This type of action reflects the manifestation of ADHD symptoms on the child’s facial expressions when unable to focus on completing the task, such as pouting, frowning, etc., and is characterized by unpredictability. It is more pronounced compared to typical children.
(5) Lying on the desk. This action is associated with symptoms of inattention. The child is unable to continue the test for an extended period, ultimately resulting in and lying on the desk.
(6) Exhibiting no significant ADHD symptoms. This state is defined as the child exhibiting normal behavior during the test, apart from the aforementioned five action states. There are no significant body or head movements.

### 3.2. Implementation Details

We divide the keypoints dataset of children with ADHD into a training set and a validation set in a 7:3 ratio to validate the effectiveness of our model. The deep learning model is implemented using PyTorch and trained on an NVIDIA RTX 4090 GPU. The training process is conducted for 100 epochs with a batch size of 8. We employ SGD with Nesterov momentum (0.9) as the optimizer, using a learning rate of 0.01 and a weight decay of 0.0001. Cross-entropy is used as the loss function.

### 3.3. Performance Comparison on SCU-ADHD-Action-Dataset

#### 3.3.1. Comparison with State-of-the-Art Models on SCU-ADHD-Action-Dataset

In this section, to verify the superiority of our network, we conduct comprehensive comparisons with state-of-the-art (SOTA) methods on the SCU-ADHD-Action-Dataset. Our comparisons encompass both skeleton-based and video-based approaches, evaluating them in terms of accuracy, computational complexity (FLOPs), and parameter counts, as shown in Table 1. The classification accuracy is calculated as:

(13) $$Acc=\frac{N_{correct}}{N_{total}}\times 100\%$$

where $N_{correct}$ represents the number of correctly classified samples and $N_{total}$ represents the total number of samples in the test set.

The FLOPs, which measure the computational complexity, is calculated as:

(14) $$FLOPs=\sum_{l=1}^{L}\left(2\times I_{l}\times O_{l}\times S_{l}\right)$$

where *L* is the total number of layers, $I_{l}$ and $O_{l}$ are the input and output channels of layer *l*, and $S_{l}$ represents the spatial dimension of the computational unit.

The total number of parameters is computed as:

(15) $$Param=\sum_{l=1}^{L}\left(I_{l}\times O_{l}\times S_{l}+O_{l}\right)$$

where the second term $O_{l}$ accounts for the bias parameters in each layer.

For skeleton-based methods, we first examine ST-GCN, which pioneered the use of GCN for action recognition. However, it achieves a relatively lower accuracy of 86.3% due to its limitation of learning only on predefined adjacency matrices. The improved AGCN enhances the performance by 2% through adaptive graph learning. MS-G3D [45] further advances the performance to 88.7% by leveraging multi-scale convolution to learn richer features. CTR-GCN [46] and Shift-GCN++ [47], two recent advanced graph convolutional networks, achieve accuracies of 88.7% and 88.9% respectively. CTR-GCN introduces dynamic topology adjustment for enhanced flexibility, while Shift-GCN++ implements a lightweight displacement-based graph convolution mechanism. SkateFormer [48], another recent transformer-based skeleton network, achieves 89.0% accuracy through its sophisticated attention mechanism.

For video-based methods, we evaluate SlowFast [49] (ResNet50 backbone) and MViTv2-S [50]. SlowFast, despite its dual-pathway architecture designed for effective temporal modeling, achieves only 68.7% accuracy on our dataset. While MViTv2-S, a multi-scale vision transformer, shows some improvement over SlowFast, both networks exhibit significantly higher computational costs. Their FLOPs are substantially higher than skeleton-based approaches, particularly MViTv2-S, which demonstrates the computational advantage of skeleton-based methods for action recognition.

Our proposed MSF-AGCN achieves better accuracy with only a modest increase in parameters compared to base AGCN. The MobileViTv2, designed for facial and head motion feature extraction, maintains exceptional efficiency with only 0.63G FLOPs. Most notably, our integrated MF-Net achieves the highest accuracy of 90.6% while maintaining reasonable computational efficiency at 6.95G FLOPs, still significantly lower than video-based alternatives.

The experimental results demonstrate that our proposed MF-Net achieves state-of-the-art performance (90.6%) on the SCU-ADHD-Action-Dataset while maintaining reasonable computational costs (6.95G FLOPs). The superior efficiency-performance trade-off of our skeleton-based approach compared to video-based methods validates the effectiveness of our multi-modal fusion strategy for ADHD action recognition.

#### 3.3.2. Comparison of Feature Extraction with t-SNE Visualization

**Figure 6:**
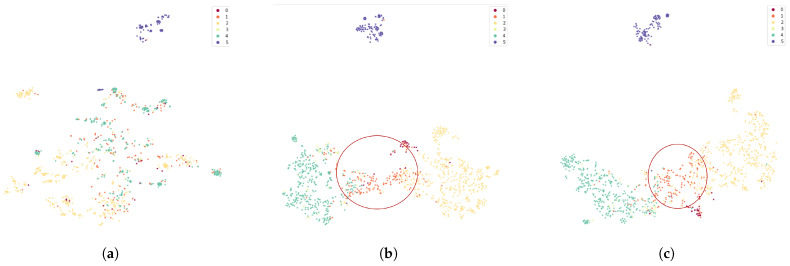
t-SNE visualization of the original data, features extracted by AGCN, and features extracted by MSF-AGCN on the validation set. The points in six colors represent the feature data of six categories. Similar feature data points are closer together, while dissimilar feature data points are farther apart. (**a**) t-SNE visualization of the original data. (**b**) t-SNE visualization of features extracted by AGCN. (**c**) t-SNE visualization of features extracted by MSF-AGCN. numbers 1–6 respectively represent the following actions. 1: Turning the head and look around. 2: Shaking body. 3: Resting head on hand. 4: Displaying facial expressions of inattention. 5: Lying on the desk. 6: Exhibiting no significant ADHD symptoms. The clustering effect of MSF-AGCN is significantly better than AGCN in the “Shaking Body” category.

We also perform t-SNE visualization of the original data, feature-extracted data by AGCN, and feature-extracted data by MSF-AGCN. t-SNE is a nonlinear algorithm that reduces high dimensional data to low-dimensional space, where similar data points are closer and dissimilar data points are farther apart. Figure 6 illustrates the clustering effects of different methods. In Figure 6a, the original data, except for class 5 (Lying on the desk) which shows clear clustering, the other five classes are more randomly distributed. In Figure 6b, the data points classified by AGCN show better clustering. In Figure 6c, MSF-AGCN demonstrates significantly better clustering than AGCN, particularly for class 1 (Shaking body, highlighted by the red circle). Overall, MSF-AGCN shows superior feature extraction for the irregular movements of ADHD children compared to AGCN.

#### 3.3.3. Validation of MSF-AGCN Module Effectiveness

**Figure 7:**
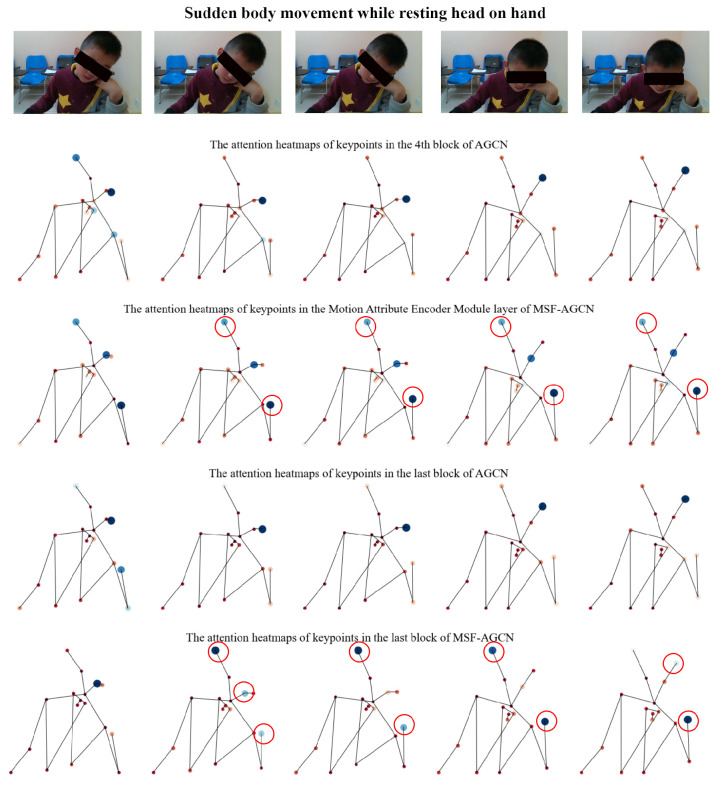
Attention heatmaps of different output layers of AGCN and MSF-AGCN during an ADHD child’s ‘sudden move while resting on his head’ action. The red circle represents the area of enhanced attention compared to the original AGCN. Our designed modules effectively help the network focus on the relevant movement areas.

In this section, to validate the effectiveness of the proposed Multi-scale Spatial-Temporal Information Extraction Module and Motion Attribute Encoder Module, we select a sudden movement action of an ADHD child to compare the performance of the AGCN and MSF-AGCN networks. Attention heatmaps are a visualization tool used to show the regions or features that a neural network model (particularly attention mechanism models) focuses on when processing input data. We compare the keypoints attention heatmaps at the same layer of AGCN without the Motion Attribute Encoder Module and the layer in MSF-AGCN where the Motion Attribute Encoder Module is placed to verify the effectiveness of this module. Additionally, we compare the keypoints attention heatmaps at the final block of MSF-AGCN, where the Multi-scale Spatial-Temporal Information Extraction Module integrates features at different scales, and the final block of AGCN to validate the effectiveness of this module. As shown in Figure 7, the top part displays frame samples of a child making a sudden move while resting on his head. Below are the attention maps between different networks and layers. From the comparison of the first two attention maps, it is evident that the MSF-AGCN network with the Motion Attribute Encoder Module can focus on areas that AGCN misses, including the hand and head. These sudden movements cause changes in the movement attributes of the ADHD child. In the keypoints heatmaps of the final output features of AGCN and MSF-AGCN, MSF-AGCN also focuses on more details of the head and hand movements in feature extraction. Overall, the two proposed modules help model some of the sudden and irregular movements of ADHD children.

#### 3.3.4. Analysis of the Impacts of Motion Attribute Encoder Module Position and Structural Graph Design

**Figure 8:**
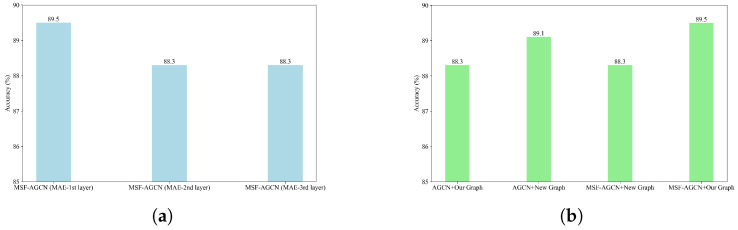
Bar chart for the Motion Attribute Encoder Module Position and different Structural Graph Designs. (**a**) Accuracy bar chart of the Motion Attribute Encoder Module placed at different stages of the layers. (**b**) Accuracy bar chart of different networks combined with different graphs.

We evaluate the performance impact of placing the Motion Attribute Encoder Module at different layers of MSF-AGCN. As illustrated in Figure 8a, when placed after the feature extraction layers of the second and third stages, the achieved accuracy rates are both 88.3%, lower than the 89.3% accuracy attained by positioning it after the first stage’s feature extraction layer. The Motion Attribute Encoder Module is designed to extract temporal attention features. An earlier integration of this module allows the model to capture salient motion cues from the more complete temporal sequences. Consequently, integrating the module at the first stage yields better performance.

We also investigate the effects of combining different networks with distinct graph representations to validate the efficacy of our proposed graph design. As shown in Figure 3, the “Our Graph” representation incorporates additional connections between the nose, mouth, and body, while the “New Graph” excludes these connections. We compare four distinct combinations: AGCN + Our Graph, MSF-AGCN + New Graph, AGCN + New Graph, and MSF-AGCN + Our Graph. As depicted in Figure 8b, the AGCN network achieves an accuracy of 89.1% when paired with the “New Graph”, outperforming its 88.3% accuracy with “Our Graph”. As a classical graph convolutional neural network, AGCN primarily focuses on modeling skeletal connections and exhibits a relatively low reliance on non-skeletal connections, such as those between limbs and the torso. Therefore, the introduction of additional non-skeletal edges in “Our Graph” may introduce certain noise, consequently impacting AGCN’s performance on this graph representation. In contrast, our proposed MSF-AGCN model, through multi-scale information extraction, demonstrates an ability to leverage the rich structural information embedded in “Our Graph”. MSF-AGCN achieves an accuracy of 89.5% when combined with “Our Graph”, outperforming its 88.3% accuracy with the “New Graph”, yielding the optimal result. This reflects that MSF-AGCN combined with “Our Graph” achieves better performance.

### 3.4. Ablation Studies

In this section, we demonstrate the effectiveness of our network through ablation experiments on different variants of AGCN and various network cues.

#### 3.4.1. Performance of MSF-AGCN

In this part, we present four variants of AGCN: the original AGCN network without any additional modules; AGCN + MSST, which includes only the Multi-scale Spatial-Temporal Information Extraction Module to verify its effect; AGCN + MAE, which includes only the Motion Attribute Encoder Module to verify its effect; and our MSF-AGCN, which integrates both the Multi-scale Spatial-Temporal Information Extraction Module and Motion Attribute Encoder Module. We conduct action recognition experiments on joints keypoints data using these four networks, and the results are shown in Table 2. The accuracy of the original AGCN on joints is 88.3% lower than the 89.5% accuracy achieved by our proposed MSF-AGCN. The accuracy of AGCN + MSST and AGCN + MAE is 89.2% and 88.7%, respectively, higher than the original AGCN, indicating the effectiveness of the MSST and MAE modules. The combined MSF-AGCN achieves the highest accuracy of 89.5%, demonstrating that combining the two modules yields the best network performance.

#### 3.4.2. Performance Evaluation of Multi-Cue Feature Fusion in MF-Net

**Figure 9:**
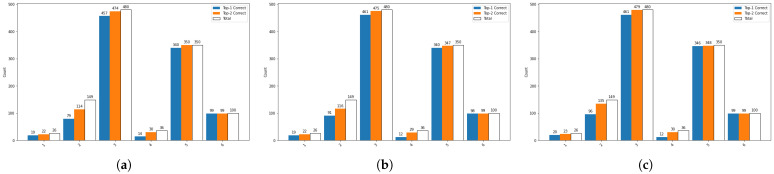
A bar chart illustrating the top-1 and top-2 counts in six categories for AGCN, MSF-AGCN, and MF-Net on the validation set. (**a**) Bar chart of top-1 and top-2 counts for six categories in AGCN. (**b**) Bar chart of top-1 and top-2 counts for six categories in MSF-AGCN. (**c**) Bar chart of top-1 and top-2 counts for six categories in MF-Net. In the chart, numbers 1–6 respectively represent the following actions. 1: Turning the head and look around. 2: Shaking body. 3: Resting head on hand. 4: Displaying facial expressions of inattention. 5: Lying on the desk. 6: Exhibiting no significant ADHD symptoms. MF-Net exhibits substantial improvements in top-1 and top-2 count classes compared to AGCN.

In this section, we first compare the action recognition accuracy on human body keypoints using MSF-AGCN for joints and bones data, MobileViTv2 for facial keypoints, and MF-Net that fuses joints, bones, and face features. As shown in Table 3, MSF-AGCN achieves an accuracy of 89.5% on joints, higher than the 88.7% on bones, because joints contain more motion information, while bones only provide relational information between joints. MobileViTv2 achieves an accuracy of 83.3% on facial keypoints, lower than on human body keypoints, as facial keypoints only provide local features and are less effective for modeling overall motion. When combining the three cues, MF-Net achieves the highest accuracy of 90.6%, indicating that integrating both global and local features from human body and facial keypoints results in the best action modeling performance. Additionally, we compare the top-1 and top-2 count class performances of MF-Net with AGCN (joints) and MSF-AGCN (joints) to highlight the performance improvements. As shown in Figure 9, MF-Net has the highest count class in five categories for both top-1 and top-2. Compared to AGCN, MF-Net improves the count class for ’Shaking body’ by 21.5%. This is due to MSF-AGCN’s better feature extraction and multi-scale feature fusion for irregular and sudden movements. Overall, MF-Net achieves top-1 and top-2 accuracies of 90.6% and 97.6%, respectively.

### 3.5. Extensive Evaluations on Public Datasets

#### 3.5.1. Public Datasets

We also select several publicly available datasets, including NW-UCLA, NTU-2D, and AFEW-VA, to evaluate our model’s performance on human body keypoints and facial keypoints.

NW-UCLA. NW-UCLA [52] dataset comprises various human actions captured from multiple camera angles. The dataset includes 10 different action categories, with each action performed by different subjects from multiple viewpoints, totaling 1494 video clips. This dataset is similar to our own, as both involve a single person performing an action. We utilize the data provided in the CTR-GCN paper, consisting of a total of 1484 files. We use data from two viewpoints as the training set and data from the remaining viewpoint as the test set to validate the performance of MSF-AGCN.

NTU-2D. The NTU-RGB+D [53] dataset is one of the most widely used datasets for skeleton-based action recognition. It includes 60 actions categorized into 40 daily activities, 9 health-related actions, and 11 mutual actions performed by pairs. The dataset comprises a total of 56,880 samples. To evaluate the performance of MSF-AGCN in recognizing actions involving multiple individuals and complex interactions, we utilize the NTU-2D dataset from MMaction. This dataset employs a keypoint extraction method similar to ours. Specifically, it uses Keypoint Fast R-CNN to extract 17 human keypoints from RGB videos. Each keypoint is characterized by three values: (x,y,s), where *x* and *y* represent the coordinates of the keypoint, and *s* indicates the confidence score of each keypoint.

AFEW-VA. AFEW-VA [54,55] (Acted Facial Expressions in the Wild-Valence and Arousal) is a facial expression video dataset specifically designed for emotion analysis and recognition. This dataset is derived from real movie clips and contains a large number of natural expressions and real-world scenes. In total, the dataset includes 600 video clips, with each frame of every video clip annotated with 68 facial landmarks. The dataset labels emotions along two dimensions: Valence and Arousal. Arousal measures the intensity or arousal level of the emotion, with low values indicating low arousal states such as calmness or fatigue, and high values indicating high arousal states such as excitement or surprise. We calculate the mean Arousal value across all frames of each video and use this mean as the label for the video. The labels are then reclassified into three categories: less than 0, 0–3, and greater than 3. To evaluate the effectiveness of MobileVitv2 in facial feature extraction, we randomly select 400 video clips as the training set and 200 video clips as the test set.

#### 3.5.2. Comparison with Some State-of-the-Art GCNs on the NW-UCLA Dataset

To validate the performance of MSF-AGCN across different datasets, we compare the performance of several state-of-the-art networks on the NW-UCLA dataset, including the ones used for our own human body keypoints dataset, with our proposed MSF-AGCN. All networks are trained using the same hyperparameters as provided in the CTR-GCN. As shown in Table 4, Shift-GCN++ achieves an accuracy of only 89.9%, which may be attributed to the small size of the dataset and the higher complexity of the model. Our MSF-AGCN shows a significant improvement over shift-GCN++, achieving an accuracy increase of 4.5%. Additionally, the performances of ST-GCN, AGCN, and CTR-GCN are similar, with accuracies of 93.1%, 93.3%, and 93.5% respectively. The MS-G3D achieves an accuracy of 94.2%. Our MSF-AGCN achieves the highest accuracy of 94.4% among the compared networks, demonstrating better action recognition capability on the NW-UCLA dataset.

#### 3.5.3. Comparison with Some State-of-the-Art GCNs on the NTU-2D Dataset

In addition to the smaller NW-UCLA dataset, we also select the larger NTU-RGB+D dataset for comparison. The NTU-RGB+D dataset is widely used for skeleton-based action recognition and contains a variety of complex actions. For this experiment, we utilize 2D keypoints data, with each frame containing 17 keypoints, similar to the keypoints data we extract in our own experiments. This consistency allows for a more direct comparison of model performance. All networks are trained using the hyperparameters provided in the CTR-GCN paper, ensuring a fair and consistent evaluation across models. As shown in Table 5, MSF-AGCN achieve an accuracy of 94.4%, a 1% improvement over AGCN. This indicates that our proposed module enhances the detail modeling of action features in AGCN, providing a more nuanced understanding of the actions. MSF-AGCN’s accuracy is second only to MS-G3D and higher than ST-GCN and AGCN, performing on par with CTR-GCN. This demonstrates that MSF-AGCN maintains strong performance even with complex actions, validating its robustness and effectiveness in action recognition tasks.

#### 3.5.4. Comparison of Different SOTA CNN Networks on Facial Keypoints Dataset and AFEW-VA Datasets

In this section, we compare the performance of several commonly used CNN classification networks, including ResNet50 [56], MobileNetV2 [57], EfficientNetV2 [58], and the network we used, MobileViTv2 [51], on facial keypoints and AFEW-VA datasets. All networks are trained with the same hyperparameters to ensure consistency. As shown in Table 6, on our own facial keypoints dataset, ResNet50, MobileNetV2, and EfficientNetV2 achieve accuracies of 81.1%, 81.7%, and 80.9%, respectively, all of which are lower than the 83.3% accuracy achieved by MobileViTv2. Similarly, on the AFEW-VA dataset, we adopt the same strategy as with the facial keypoints dataset, constructing 68×60 facial keypoints images for each video. This dataset only classifies the emotional intensity of the face. The accuracies of ResNet50, MobileNetV2, and EfficientNetV2 on this dataset are 58.5%, 62.5%, and 62.5%, respectively. MobileViTv2 achieved an accuracy of 65.5%, outperforming the three CNN networks. MobileViTv2 also has fewer parameters compared to ResNet50 and EfficientNetV2. Therefore, whether on our own dataset or on the AFEW-VA dataset, MobileViTv2 demonstrates better modeling capability for facial features.

## 4. Conclusions

In this paper, we present a solution for recognizing behavioral actions of children with ADHD during the TOVA visual assessment, aiming to assist clinicians in symptom evaluation. To achieve this goal, we propose a Multi-cue Feature Fusion Network (MF-Net) based on keypoint representations. Our method encompasses collecting behavioral videos of ADHD children during the TOVA test and extracting human body and facial keypoints. For the human body keypoints, We introduce a novel Multi-scale Features and Frame-Attention Adaptive Graph Convolutional Network (MSF-AGCN) to extract global motion features from joints and bones. For the facial keypoints, we transform them into image representations and employ the MobileVitv2 model for feature extraction. The features from these three cues are then integrated to yield the final action recognition result. Experimental results on both our dataset and public benchmarks demonstrate promising action recognition accuracy, validating the effectiveness of our approach.

Our work introduces several innovative technical contributions tailored for effective ADHD behavior recognition during TOVA assessments. The proposed MSF-AGCN represents a novel approach to extract global motion features from irregular and impulsive movements exhibited by ADHD children. Specifically, the Multi-scale Spatial-Temporal Information Extraction Module enables comprehensive temporal feature extraction by addressing challenges unique to ADHD-specific behavioral patterns. Moreover, the Motion Attribute Encoder Module introduces an new temporal attention mechanism designed to identify diagnostically relevant motion attributes. Furthermore, our multi-cue fusion strategy presents an innovative approach to combining global body motion features with local facial and head movement characteristics, allowing for a more comprehensive extraction of clinically meaningful behavioral cues.

In conclusion, our work contributes a novel approach to ADHD action recognition during TOVA assessments, providing clinicians with a valuable, quantitative tool for evaluating ADHD symptoms and monitoring treatment efficacy and patient progress over time. While our current results are promising, future work should focus on expanding the dataset to address class imbalance issues, and possibly incorporating additional modalities to further optimize the network’s performance and robustness in real-world clinical settings, including evaluating the impact of adversarial examples on model performance, similar to their use in image datasets as discussed in recent studies [59]. 

## Figures and Tables

**Table 1:** Comparison of Accuracy on SCU-ADHD-Action-Dataset with State-of-the-Art Models.

Methods	Accuracy (%)	FLOPs (G)	#Param (M)
ST-GCN [39]	86.3	2.22	3.
AGCN [40]	88.3	2.54	3.44
MS-G3D [45]	88.7	3.50	2.72
CTR-GCN [46]	88.7	1.12	1.41
Shift-GCN++ [47]	88.9	0.42	0.45
SkateFormer [48]	89.0	1.95	3.31
SlowFast [49]	68.7	13.91	33.57
MViTv2-S [50]	86.7	99.0	34.07
MSF-AGCN	89.5	3.16	3.91
MF-Net (proposed)	90.6	6.95	17.61

**Table 2:** Comparison of Accuracy on SCU-ADHD-Action-Dataset among Different AGCN Variants.

Methods	Accuracy-Joints (%)
AGCN [40]	88.3
AGCN + MSST	89.2
AGCN + MAE	88.7
MSF-AGCN	89.5

**Table 3:** Comparison of Accuracy on SCU-ADHD-Action-Dataset among Different Network Cues.

Methods	Accuracy (%)
MSF-AGCN (joints)	89.5
MSF-AGCN (bones)	88.7
MobileViTv2 [51] (face)	83.3
MF-Net (joints + bones + face)	90.6

**Table 4:** Comparison of Accuracy on NW-UCLA Dataset with Some State-of-the-Art GCNs.

Method	Accuracy (%)
ST-GCN [39]	93.1
AGCN [40]	93.3
MS-G3D [45]	94.2
CTR-GCN [46]	93.5
Shift-GCN++ [47]	89.9
MSF-AGCN	94.4

**Table 5:** Comparison of Accuracy on NTU-2D Dataset with Some State-of-the-Art GCNs.

Method	Accuracy (%)
ST-GCN [39]	93.9
AGCN [40]	93.4
MS-G3D [45]	94.8
CTR-GCN [46]	94.3
MSF-AGCN	94.4

**Table 6:** Comparison of Different SOTA CNN Networks on Facial Keypoints and AFEW-VA Datasets.

Methods	Facial Keypoints Dataset (%)	AFEW-VA (%)	Param.
ResNet50 [56]	81.1	58.5	27.5 M
MobileNetV2 [57]	81.7	62.5	1.5 M
EfficientNetV2 [58]	80.9	62.5	48.2 M
MobileViTv2 [51]	83.3	65.5	10.6 M

## Data Availability

The data will be available upon reasonable request from the corresponding author.
